# Possible Uses of Plants of the Genus *Asphodelus* in Oral Medicine

**DOI:** 10.3390/biomedicines7030067

**Published:** 2019-09-02

**Authors:** Mario Dioguardi, Pierpaolo Campanella, Armando Cocco, Claudia Arena, Giancarlo Malagnino, Diego Sovereto, Riccardo Aiuto, Luigi Laino, Enrica Laneve, Antonio Dioguardi, Khrystyna Zhurakivska, Lorenzo Lo Muzio

**Affiliations:** 1 Department of Clinical and Experimental Medicine, University of Foggia, Via Rovelli 50, 71122 Foggia, Italy; pierpaolo.campanella78@gmail.com (P.C.); armando.cocco@gmail.com (A.C.); claudia.arena@unifg.it (C.A.); giancarlomalagnino@gmail.com (G.M.); diego_sovereto.546709@unifg.it (D.S.); enrica.laneve@unifg.it (E.L.); antoniodioguardi@gmail.com (A.D.); khrystyna.zhurakivska@unifg.it (K.Z.); lorenzo.lomuzio@unifg.it (L.L.M.); 2 Department of Biomedical, Surgical, and Dental Science, University of Milan, 20122 Milan, Italy; Riccardo.Aiuto@unimi.it; 3 Multidisciplinary Department of Medical-Surgical and Odontostomatological Specialties, University of Campania “Luigi Vanvitelli”, 80121 Naples, Italy; luigi.laino@unicampania.it

**Keywords:** *Asphodelus microcarpus*, *Asphodelus tenuifolius*, *Asphodelus aestivus*, oral medicine, medical plants, ethnomedicine, skin diseases

## Abstract

Among the many plants used in traditional medicine we have the plants of the genus *Asphodelus*, which are present in the Mediterranean area in North Africa and South East Asia, and have been used by indigenous peoples until recently for various pathologies, including: Psoriasis, alopecia areata, acne, burns, nephrolithiasis, toothache, and local inflammation. The scientific literature over the last five years has investigated the various effects of the metabolites extracted from plants of the genus *Asphodelus*, paying attention to the diuretic, antihypertensive, antimicrobial, anti-inflammatory, and antioxidant effects, and it also has begun to investigate the antitumor properties on tumor cell lines. Studies have been identified through bibliographic research on electronic databases. A total of 574 records were identified on the PubMed, Scopus, Web of Science, and EBSCO databases. After having proceeded to the screening of the articles with the application of the eligibility criteria (all the articles pertaining to the issue *Asphodelus*), we arrived at a number of 163 articles, and then after the elimination of overlaps, to 82 articles. There are 11 articles which investigate the possible uses of plants of the genus *Asphodelus* in oral medicine. In oral medicine, the possible uses investigated by the scientific literature are for the treatment of neoplastic (melanoma and oral cancer), viral (herpetic viruses), and microbial diseases (candida, bacteriosis, leishmaniasis), and in the affection of the skin.

## 1. Introduction

The search for new active principles, which can be used in modern medicine, leads researchers to turn their attention to the plants that are part of traditional medicine, and to the discovery of new substances at the base of the alleged therapeutic effects [1]. However, there is not always a comparison between the traditional use of a plant in the treatment of a disease and their real action when investigated experimentally and clinically.

Among the many plants used in traditional medicine, we have the plants of the genus *Asphodelus,* which are present in the Mediterranean area in North Africa and South East Asia, and have been used by indigenous peoples until recently for various pathologies, including: Psoriasis, alopecia areata [2], acne [3], burns, nephrolithiasis, toothache, and local inflammation.

**Figure 1:**
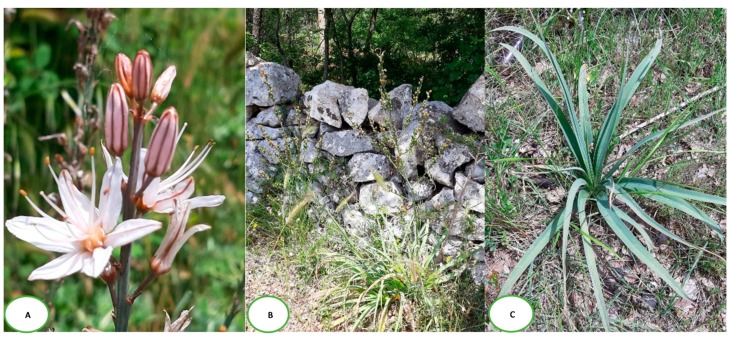
*Asphodelus microcarpus*: (**A**) Flower; (**B**) plant with fruits; (**C**) leaves; Photographs taken in the Pianelle woods, Martina Franca, Italy, May 2019.

Eighteen species belong to the genus *Asphodelus,* with different sub-species and varieties, and those most described are the species *Asphodelus tenuifolius*, *Asphodelus microcarpus*, *Asphodelus ramosus*, *Asphodelus aestivus* (the names *microcarpus*, *ramosus*, and *eastivus* indicate the same species of *Asphodelus*), and *Asphodelus fistulosus*. The anatomical parts used of the plant are: The bulbs, roots, stem, leaves, and seeds, as decoctions to be applied on the afflicted areas both as extracts in aqueous and alcoholic solutions. Moreover, the plants of the *Asphodelus* are consumed as foods in the gastronomic tradition of some countries (Figure 1). 

A previous review conducted by Malmir et al. (2018) focused on this plant by investigating the main metabolites derived from the genus *Asphodelus* and their bioactivity, by searching the various metabolites obtained in the literature [4].

The scientific literature over the last five years has investigated the various effects of the metabolites extracted from plants of the genus *Asphodelus*, paying attention to the diuretic, antihypertensive [5], antimicrobial [6], anti-inflammatory, and antioxidant effects [7], and it also has begun to investigate the anticancer properties on tumor cell lines [8].

Indeed, recent research conducted by Khalfaoui et al. (2018) investigates the antitumor properties of a metabolite (glucopyranosylbianthrone), extracted from the species *Asphodelus tenuifolius*, directed toward melanoma cells, giving further interest to the genus *Asphodelus* [9].

Furthermore, a study conducted by Mayouf et al. (2019) confirms the activity of the metabolites extracted from *Asphodelus microcarpus* as an antioxidant and anti-inflammatory, partially confirming its use in traditional medicine as an anti-inflammatory [7].

From these studies we can see, in the field of oral pathology, the possible uses of the substances extracted not only as antimicrobial and anti-inflammatory agents, but also as antitumors, both for an antioxidant action and for a direct action against some tumor cell lines.

In the light of the growing interest in medicine in the search for pharmacologically active substances, the question we ask ourselves is: What are the real, possible, and potential uses of plants of the genus *Asphodelus* in the treatment of diseases affecting the oral cavity and the maxillofacial district, transcending the uses of present folkloristic medicine, that have been described in the literature?

## 2. Materials and Methods

The potentially eligible studies are literature reviews, clinical studies, in vitro studies, and epidemiological studies that treat *Asphodel* and its metabolites in modern medicine, and especially in maxillofacial disorders. All the studies published in English and conducted over the last 50 years have been taken into consideration, with particular attention in the last decade, considering the growing interest in the rediscovery of natural principles present in folkloristic medical cultures.

The potentially eligible articles were subjected to a full text analysis to verify their use for a qualitative analysis.

- The inclusion criteria applied for the quantitative analysis are to include all those studies that spoke of asphodel in the medical field.
- The exclusion criteria are to exclude all those studies that do not deal with asphodel for a potential use of its metabolites in the field of medicine and oral diseases, or for the diseases that affect the maxillofacial district.

Studies have been identified through bibliographic research on electronic databases [10].

The literature search was conducted on the PubMed, Scopus, Web of Science, and EBSCO databases. The search for providers was conducted between 25 May 2019 and 10 June 2019, and the last search for a partial update of the literature was conducted on 20 June 2019.

The following search terms were used on PubMed, Scopus, Web of Science, and EBSCO: PubMed *asphodelus* 46 records, *Asphodelus ramosus* 5 records, *Asphodelus microcarpus* 16 records, *Asphodelus tenuifolius* 10 records, *Asphodel* 18 records, *Asphodelus aestivus* 4 records, Scopus *asphodelus* 255 records, EBSCO *asphodelus* 46 records, Web of science *asphodelus* 174 records (Table 1). 

Two reviewers were appointed to identify and screen the records, and a third reviewer decided in doubtful situations. After the screening phase, the overlaps were removed, the studies were identified, and the choice of studies was included in the qualitative analysis.

## 3. Results

A total of 574 records were identified on the PubMed, Scopus, Web of Science, and EBSCO databases (Table 1).

After having proceeded to the screening of the articles, with the application of the eligibility criteria (all the articles that speak of the *Asphodelus* as a possible employment in the medicine), we arrive at 163 articles, with the elimination of the overlaps to 82 articles, which with the application of the inclusion and exclusion criteria leads to 11 studies that treat *Asphodelus* as a possible use in oral medicine.

**Figure 2:**
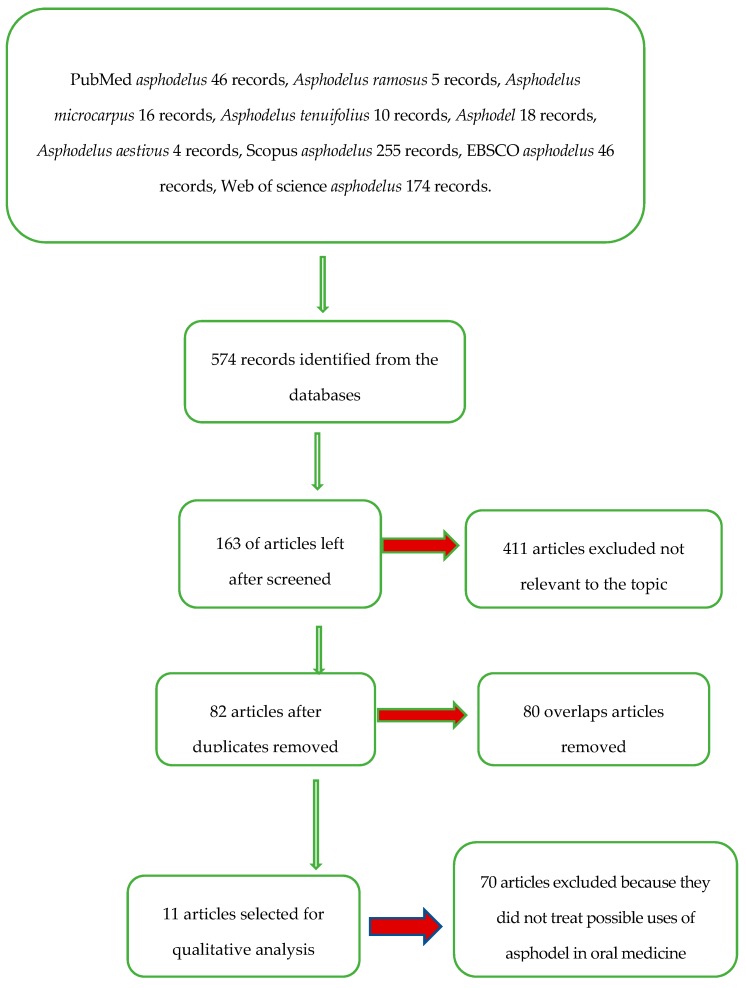
Flow chart of the different phases of the review.

The whole selection and screening procedure, as described in Table 1, is represented in the flow chart (Figure 2).

The eleven studies selected at the end of the bibliographic research phase were reported in Table 2. The bibliographic data, the variety of asphodel studied, the type of extract used, and the part of the plant used were reported. Furthermore, the results of the single studies and the indications on the possible uses in oral medicine have been reported.

## 4. Discussion

The analysis of the literature shows that the extracts of the plant of the genus *Asphodelus* have been used and tested for the treatment of various pathologies, among which: Hypertension for its vasodilatory and diuretic effects, as reported by Aslam et al. (2016) [19] on a study on mice reporting a vasodilator effect due to a mechanism similar to calcium channel blockers, and a diuretic effect similar to thiazides; for the treatment of gastric ulcer, Gürbüz et al. (2002) shows a use of the root of *Asphodelus eastivus* with a gastro-protector effect [20]; Anand et al. 2012 [21] and Sharma et al. (2011) report a medical use, the first in Africa and the second in India, for the treatment of nephrolithiasis with an increase in diuresis [22].

In addition, plant extracts of the genus *Asphodelus*, both in alcoholic and aqueous solutions, have been tested for their antibacterial properties, sometimes reporting conflicting results, as in the study by Sibanda et al. (2007) which evaluated the extracted Asphodelin A activity from *Asphodelus microcarpus* as an adjuvant in antibiotic therapies with no efficacy [23].

The antioxidant properties of the metabolites extracted from *Asphodelus* are also widely known as reported by Younis et al. (2017) [5] for *Asphodelus tenuifolius,* and Ljubuncic et al. (2005) for *Asphodelus microcarpus* [24].

### 4.1. Possible Uses of plants of the Genus Asphodelus in Oral Medicine

In the diseases of the maxillofacial district, the possible uses investigated by the scientific literature, transcending the traditional uses of medicine in the various cultures, are in the treatment of neoplastic (melanoma and oral cancer), viral (herpetic viruses), and microbial diseases (candida, bacteriosis, leishmaniasis), and in the affection of the skin.

#### 4.1.1. Anticancer, Antioxidant, and Anti-Inflammatory Properties

Plants of the genus *Asphodelus* have been studied for their metabolites for anticancer properties, both directed in adjuvant therapies in oral cancer, and directly on melanoma cell lines.

The antitumor activity directed against melanoma cells is mainly based on the properties of the anthraquinones, extracted from this plant, in presenting cytotoxicity.

Indeed, Khalfaoui et al. report interesting data on the two metabolites (glucopyranosylbianthrones, two atropisomeric forms) asphodeline 1 and 2, extracted in alcoholic solution of the aerial parts of *Asphodelus tenuifolius* These metabolites showed a cytotoxic activity toward melanoma cell lines (A375 cells), and both asphodeline inhibited cell proliferation in a concentration-dependent manner, with IC_50_ values of 20.6 ± 0.8 and 23.2 ± 1.1 μM, respectively. According to this study, the targets in expressing their cytostatic and cytotoxic action against melanoma, are: Adenosine A_2a_ (the antagonist plays an important role in inhibiting promoter effect in melanoma tumor tissue); inhibition of glycogen synthase kinase (GSK)-3β, linked to the reduction of melanoma invasiveness; and inhibition of the Polo-like kinase 1 (PLK1), that the expression is dynamically regulated during the cell cycle in melanoma cells [9].

The study by Di Petrillo et al. in 2016 is also interesting, which identifies in the extracts in alcohol, methanol, and water (flower, leaves, and tuber) of the *Asphodelus microcarpus* substances active against anti-tyrosinase, which is a key enzyme in melanin production [11].

The study demonstrates an anti-melaninogenic effect aimed at the B16F10 cells murine melanoma cells. Furthermore, the best results were obtained mainly with the extract of *Asphodelus microcarpus* flowers.

In a study conducted in 2011, Phangani reported an antimicrobial activity of the fruits of *Aspodelus tenuifolius* turned toward bacteria and fungi, cultivated starting from the swabs of 40 patients in radiotherapy treatment for oral carcinoma. Thus, placing as a possible adjuvant therapy of oral carcinoma, in the ability of the active ingredients present in *Asphodelus* to inhibit bacterial proliferation in patients with oral cancer [12].

Furthermore, according to Mayouf et al., which confirms numerous previous studies, the extract of *Asphoelus microcarpus* has anti-inflammatory and antioxidant properties [7]. The power to turn off the inflammatories, and the antioxidant capacity of this plant, make it a potential weapon for reducing risk factors for the development of neoplastic lesions [25].

A possible use of *Asphodelus* extracts for acute chronic inflammatory diseases (local inflammation in the course of stomatitis, or acute and chronic periapical endodontic lesions) are described by Mayouf et al. in 2018 [7]. The experiment was carried out on rats and mice with extracts in alcoholic solution (methanol) of aerial parts and roots of *Asphodelus microcarpus,* giving statistically significant results in the second phase of inflammation (3–6 h), with an effect similar to diclofenac.

#### 4.1.2. Antiviral, Antifungal, and Antibacterial Properties

Numerous studies have investigated the properties of *Asphodelus* extracts on the activity of microorganisms. In this review, we have only taken into consideration the studies concerning pathogens involved in oral cavity stomatitis and skin infections.

Confirmation of the antibacterial activity of *Asphodelo* can be found in the Di Petrillo et al. (2017) study, which indicates the ethanolic extract of *Asphodelus microcarpus* as an inhibitor of the activity of *Escherichia coli, Staphylococcus aureus,* and of *Candida albicans* [13].

Furthermore, *Staphylococcus aureus* was the subject of a study by Al-Kayali et al. (2016) [14], where the bacterium was inhibited with an area of 18.6 mm diameter by the raw bulb extract and aerial part of *Asphodelus microcarpus*.

The activity of *Asphodelus microcarpus* extracts against *Staphylococcus aureus* is therefore confirmed in two studies; moreover, the methanol extract of *Asphodelus tenuifolius* was found to have an inhibiting effect with a diameter of 16 mm against *Staphylococcus aureus*, with a dosage of 4 mg/mL (Eddine et al. 2015) [15].

In addition, a study conducted by Fafal et al. in 2016 reports that *Asphodelus aestivus* oil showed moderate antibacterial activity against *Staphylococcus aureus*, *Staphylococcus epidermidis, Escherichia coli*, *Klebsiella pnemoniae*, and *Pseudomonas aeruginosa,* and also showed antifungal activity against *Candida albicans* [16].

The ability of *Staphylococcus aureus* to determine stomatitis is known in the literature [26,27,28], and it is therefore essential in oral medicine to identify new active ingredients aimed at this bacterium, having in recent decades developed resistance to common antibiotics.

The aerosol parts of *Asphodelus microcarpus* have been tested against acne vulgaris bacteria (*Propionibacterium acnes*) demonstrating efficacy, according to Nelson et al. (2016) [3].

An effect against the protozoa of leishmaniosis has been investigated by EL-ON et al. (2009), without showing particularly effective results [17]. Abad et al. (2000) tested the alcohol extract of *Asphodelus ramosus* as an antiviral extract, against *Herpes simplex* type I (HSV-1), *Vesicular stomatitis virus* (VSV), and *Poliovirus* type 1, reporting these data as ineffective [18]; however, these data apparently appear to be in contrast with those of Di Petrillo et al. of 2017, which instead noted an effect of the *Asphodelus microcarpus* extract, which significantly affected the Ebola virus VP35 inhibition of the viral RNA (vRNA) induced IFN response [13].

## 5. Conclusions

In scientific literature, the plants of the genus *Asphodelus* that can potentially be used in oral medicine are *Asphodelus tenuifolius* and *Asphodelus microcarpus (ramosus* and *eastivus* are synonyms).

The extracts of both plants have an antibacterial activity especially against the *Staphylococcus aureas* (responsible for stomatitis and tonsillitis), with an antifungal activity against *Candida albicans* (oral candidiasis).

For Hsv1 (herpetic virus) the inhibitory activity was modest, but according to the authors not enough to justify a clinical use.

The antioxidant and anti-inflammatory properties of the genus *Asphodelus* are interesting for their uses as protective factors against neoplasms; moreover, they are interesting for their cytotosic effect against the melanoma cell line (A375 cells) and the inhibiting activity against the tyrosine kinase in the production of melanin.

So, the possible and future uses of *Asphodelus* extracts in the field of oral medicine may be:

- As an anti-inflammatory aimed at inflammatory diseases of the oral cavity and skin;
- Anti-acne due to its inhibitory activity toward *Propionibacterium acnes*;
- As an antibacterial agent for stomatitis and bacterial inflammation (Staphylococcus aureus, Staphylococcus epidermidis, Escherichia coli, Klebsiella pnemoniae, and Pseudomonas aeruginosa);
- As antiviral (HSV1) and anti-protozoa (leishmaniosis);
- In the treatment of oral candidiasis for its antifungal properties;
- In the treatment of neoplasms (cytotoxic action directed toward melanoma cells, in vitro studies);
- As an antioxidant.

## Figures and Tables

**Table 1:** Complete overview of the search methodology. Records identified by databases: 574; articles selected for qualitative analysis: 11.

Data Base	Search Term	Records	Selected Records	Removal Overlaps	Articles Concerning the Pathologies of the Oro-Maxillo Facial Area
PubMed	*asphodelus*	46	23	\	\
PubMed	*Asphodelus ramosus*	5	2	\	\
PubMed	*Asphodelus microcarpus*	16	13	\	\
PubMed	*Asphodelus tenuifolius*	10	6	\	\
PubMed	*Asphodel*	18	3	\	
PubMed	*Asphodelus aestivus*	4	1	\	\\
Scopus	*asphodelus*	255	55	\	\
EBSCO	*asphodelus*	46	24	\	\
Web of Science	*asphodelus*	174	36	\	\
Total		574	163	82	11

**Table 2:** The main characteristics of the 11 selected studies are described, with reference to the authors, the year of publication, the variety of *Asphodel*, the type of extract, and the results obtained.

Author, Data, Journal	Type of Study	Type of Asphodel	Part of the Plant Investigated	Type of Extract	Active Principles Investigated or Identified	Pathologies and Effects Studied	Animals, Cell Lines, Microorganism on Which the Extract Was Tested	Indications on Possible Uses in Medicine and Oral Pathologies	Results
Khalfaoui et al. 2018, J Nat Prod [9]	Vitro	*Asphodelus tenuifolius*	Extract of the aerial part	-	Glucopyranosylbianthrones (1 and 2)	Human melanoma	Human melanoma A375 cells	Oral melanoma	Inhibition of melanoma cell proliferation
Di Petrillo et al. 2016, BMC Complement Altern Med [11]	Vitro	*Asphodelus microcarpus*	Flower, leaves, and tuber	Extracts in alcohol, methanol, and water	Luteolin	Pigmentation disorders	Melanoma murin B16F10 cells	Pigmentation disorders	Tyrosinase inhibitory activity
Panghal et al. 2011, Ann Clin Microbiol Antimicrob [12]	Vitro	*Asphodelus tenuifolius*	Fruits	-	Alkaloid, anthraquinones, reducing sugars, tannins, steroids.	Antimicrobial activity in patients with oral cancer	Oral microorganisms (salivary swabs of patients with oral cancer)	Adjuvant treatments in the treatment of oral infections in patients with oral cancer	Inhibit bacterial proliferation in patients with oral cancer
Mayouf et al. 2019, J Ethnopharmacol [7]	Vitro/vivo	*Asphodelus microcarpus*	Aerial part, leaf, stem flowers, and root.	Extracts in methanol	Polyphenols and flavonoids	Antioxidant and anti-inflammatory properties	Mice	Possible to use in the treatment of inflammatory processes of the oral cavity	Anti-inflammatory and antioxidative effect
Di Petrillo et al. 2017, BMC Microbiol [13]	Vitro	*Asphodelus microcarpus*	Leaves	Extract in ethanol	Polyphenols and flavonoids	Antiviral, antibiotic and antifungal properties	A549 cells, Gram (+) Bacteria, Gram (−) Bacteria, Candida albicans,	Possible to use in the treatment of oral bacterial infections supported by Gram (+), Gram (–), and in the treatment of oral candidiasis	Inhibitor of the activity of *Escherichia coli*, *Staphylococcus aureus* and of *Candida albicans*
Al-kayali et al. 2016, IJPPR [14]	Vitro	*Asphodelin lutea and Asphodelus microcarpus*	Aerial parts and bulbs	Extracts in alcohol, methanol, and water	1,8-dihydroxyanthraquinones	Antibiotic properties	Methicillin Resistant *Staphylococcus aureus*	Oral infections sustained by staphylococci	Inhibitor of the activity *Staphylococcus aureus*
Eddine et al. 2015, IJPCR [15]	Vitro	*Asphodelus tenuifolius*	Aerial parts	Extracts in alcohol, methanol, and petroleum ether	Glycosides, anthraquinones, flavonoids, steroids, proanthocyanidins, tanninis, Phenolic compound	Antibiotic and antioxidant properties	*Staphylococcus* aureus	In the treatment of oral bacterial infections and as an antioxidant in preventing the action of free radicals	Antioxidant and antibacterial activities
Fafal et al. 2016, Human and Veterinary Medicine [16]	Vitro	*Asphodelus aestivus*	Seeds	The oil extraction of dried and powdered seeds	Fatty acid	Antimicrobial activities and antifungal proprieties	Gram (+) Bacteria, Gram (−) Bacteria. Candida albicans	Possible use in the treatment of oral bacterial infections supported by Gram (+), Gram (–), and in the treatment of oral candidiasis	Moderate antimicrobial activity against Gram (+), Gram (−) bacteria
Nelson et al. 2016, Front Pharmacol [3]	Vitro	*Asphodelus microcarpus*	Infructescence, leaves.	Crude extracts	-	Acne	Propionibacterium acnes	Acne treatment	Growth inhibitory activity
El-On et al. 2009, Ann Trop Med Parasitol [17]	Vitro, vivo	*Asphodelus ramosus*	Leaves	Extracts in methanol	-	Antileishmanial activity	Male C3H/HeJ mice, Leishmania parassite	Possible use in the treatment of leishmaniasis	No effectiveness
Abad et al. 2000, Phytother Res [18]	Vitro	*Asphodelus ramosus*	-	Alcohol Ethanol and Aqueous extract	-	Anti-viral activity	(HSV-1, VSV, and poliovirus type 1)	Possible use in the treatment of oral herpes lesions	No effectiveness

## References

[1] Perrone D., Ardito F., Giannatempo G., Dioguardi M., Troiano G., Lo Russo L., De Lillo A., Laino L., Lo Muzio L. (2015). Biological and therapeutic activities, and anticancer properties of curcumin. Exp. Ther. Med..

[2] Rezghi M., Fahimi S., Zakerin S. (2016). The most frequent herbs proposed by iranian traditional medicine for alopecia areata. Iran. J. Med. Sci..

[3] Nelson K., Lyles J.T., Li T., Saitta A., Addie-Noye E., Tyler P., Quave C.L. (2016). Anti-acne activity of italian medicinal plants used for skin infection. Front. Pharmacol..

[4] Malmir M., Serrano R., Canica M., Silva-Lima B., Silva O. (2018). A comprehensive review on the medicinal plants from the genus asphodelus. Plants.

[5] Younis W., Alamgeer, Schini-Kerth V.B., Junior A.G., Majid M. (2018). Cardioprotective effect of asphodelus tenuifolius cav. On blood pressure and metabolic alterations in glucose-induced metabolic syndrome rats-an ethnopharmacological approach. J. Ethnopharmacol..

[6] Salhi N., Mohammed Saghir S.A., Terzi V., Brahmi I., Ghedairi N., Bissati S. (2017). Antifungal activity of aqueous extracts of some dominant algerian medicinal plants. BioMed Res. Int..

[7] Mayouf N., Charef N., Saoudi S., Baghiani A., Khennouf S., Arrar L. (2019). Antioxidant and anti-inflammatory effect of asphodelus microcarpus methanolic extracts. J. Ethnopharmacol..

[8] Zhurakivska K., Troiano G., Caponio V.C.A., Dioguardi M., Arena C., Lo Muzio L. (2018). The effects of adjuvant fermented wheat germ extract on cancer cell lines: A systematic review. Nutrients.

[9] Khalfaoui A., Chini M.G., Bouheroum M., Belaabed S., Lauro G., Terracciano S., Vaccaro M.C., Bruno I., Benayache S., Mancini I. (2018). Glucopyranosylbianthrones from the algerian asphodelus tenuifolius: Structural insights and biological evaluation on melanoma cancer cells. J. Nat. Prod..

[10] Lo Russo G., Spolveri F., Ciancio F., Mori A. (2013). Mendeley: An easy way to manage, share, and synchronize papers and citations. Plast. Reconstr. Surg..

[11] Di Petrillo A., Gonzalez-Paramas A.M., Era B., Medda R., Pintus F., Santos-Buelga C., Fais A. (2016). Tyrosinase inhibition and antioxidant properties of asphodelus microcarpus extracts. BMC Complement. Altern. Med..

[12] Panghal M., Kaushal V., Yadav J.P. (2011). In Vitro antimicrobial activity of ten medicinal plants against clinical isolates of oral cancer cases. Ann. Clin. Microbiol. Antimicrob..

[13] Di Petrillo A., Fais A., Pintus F., Santos-Buelga C., Gonzalez-Paramas A.M., Piras V., Orru G., Mameli A., Tramontano E., Frau A. (2017). Broad-range potential of asphodelus microcarpus leaves extract for drug development. BMC Microbiol..

[14] Al-kayali R., Haroun M.F., Kitaz A. (2016). Antibacterial activity of asphodelin lutea and asphodelus microcarpus against methicillin resistant staphylococcus aureus. Int. J. Pharmacog. Phytochem. Res..

[15] Eddine S.E., Segni L., Ridha O.M. (2015). In Vitro assays of the antibacterial and antioxidant properties of extracts from asphodelus tenuifolius cav and its main constituents: A comparative study. Int. J. Pharm. Clin. Res..

[16] Fafal T., Yilmaz F.F., Birincioğlu S.S., Hoşgör-Limoncu M., Kivçak B. (2016). Fatty acid composition and antimicrobial activity of asphodelus aestivus seeds. Hum. Vet. Med..

[17] El-On J., Ozer L., Gopas J., Sneir R., Enav H., Luft N., Davidov G., Golan-Goldhirsh A. (2009). Antileishmanial activity in israeli plants. Ann. Trop. Med. Parasitol..

[18] Abad M.J., Guerra J.A., Bermejo P., Irurzun A., Carrasco L. (2000). Search for antiviral activity in higher plant extracts. Phytother. Res..

[19] Aslam N., Janbaz K.H., Jabeen Q. (2016). Hypotensive and diuretic activities of aqueous-ethanol extract of asphodelus tenuifolius. Bangladesh J. Pharmacol..

[20] Gurbuz I., Ustun O., Yesilada E., Sezik E., Akyurek N. (2002). In Vivo gastroprotective effects of five turkish folk remedies against ethanol-induced lesions. J. Ethnopharmacol..

[21] Tiwari A., Soni V., Londhe V., Bhandarkar A., Bandawane D., Nipate S. (2012). An overview on potent indigenous herbs for urinary tract infirmity: Urolithiasis. Asian J. Pharm. Clin. Res..

[22] Sharma N., Tanwer B.T., Vijayvergia R. (2011). Study of medicinal plants in aravali regions ofrajasthan for treatment of kidney stone and urinary tract troubles. Int. J. PharmTech Res..

[23] Sibanda T., Okoh A.I. (2007). The challenges of overcoming antibiotic resistance: Plant extracts as potential sources of antimicrobial and resistance modifying agents. Afr. J. Biotechnol..

[24] Ljubuncic P., Azaizeh H., Portnaya I., Cogan U., Said O., Abu Saleh K., Bomzon A. (2005). Antioxidant activity and cytotoxicity of eight plants used in traditional arab medicine in israel. J. Ethnopharmacol..

[25] Subapriya R., Kumaraguruparan R., Nagini S., Thangavelu A. (2003). Oxidant-antioxidant status in oral precancer and oral cancer patients. Toxicol. Mech. Methods.

[26] Dioguardi M., Di Gioia G., Illuzzi G., Arena C., Caponio V.C.A., Caloro G.A., Zhurakivska K., Adipietro I., Troiano G., Lo Muzio L. (2019). Inspection of the microbiota in endodontic lesions. Dent. J..

[27] Shouval D.S., Bilavsky E., Avitzur Y., Shapiro R., Amir J. (2008). Staphylococcus aureus bacteremia complicating herpes simplex virus type 1 stomatitis: Case report and review of the literature. J. Periodontol..

[28] Garbacz K., Jarzembowski T., Kwapisz E., Daca A., Witkowski J. (2019). Do the oral Staphylococcus aureus strains from denture wearers have a greater pathogenicity potential?. J. Oral Microbiol..

